# SHVC Tile-Based 360-Degree Video Streaming for Mobile VR: PC Offloading Over mmWave

**DOI:** 10.3390/s18113728

**Published:** 2018-11-01

**Authors:** Dien Van Nguyen, Tuan Thanh Le, Sangsoon Lee, Eun-Seok Ryu

**Affiliations:** Department of Computer Engineering, Gachon University, 1342 Seongnamdaero, Sujeong-gu, Seongnam, Gyeonggi 1320, Korea; diennv1990@gc.gachon.ac.kr (D.V.N.); tuanlt@gc.gachon.ac.kr (T.T.L.)

**Keywords:** PC offloading, mmWave, SHVC, parallel video processing, mobile virtual reality

## Abstract

360-degree video streaming for high-quality virtual reality (VR) is challenging for current wireless systems because of the huge bandwidth it requires. However, millimeter wave (mmWave) communications in the 60 GHz band has gained considerable interest from the industry and academia because it promises gigabit wireless connectivity in the huge unlicensed bandwidth (i.e., up to 7 GHz). This massive unlicensed bandwidth offers great potential for addressing the demand for 360-degree video streaming. This paper investigates the problem of 360-degree video streaming for mobile VR using the SHVC, the scalable of High-Efficiency Video Coding (HEVC) standard and PC offloading over 60 GHz networks. We present a conceptual architecture based on advanced tiled-SHVC and mmWave communications. This architecture comprises two main parts. (1) Tile-based SHVC for 360-degree video streaming and optimizing parallel decoding. (2) Personal Computer (PC) offloading mechanism for transmitting uncompressed video (viewport only). The experimental results show that our tiled extractor method reduces the bandwidth required for 360-degree video streaming by more than 47% and the tile partitioning mechanism was improved by up to 25% in terms of the decoding time. The PC offloading mechanism was also successful in offloading 360-degree decoded (or viewport only) video to mobile devices using mmWave communication and the proposed transmission schemes.

## 1. Introduction

Recently, 360-degree video streaming for virtual reality (VR) that is currently available on some major video platforms, such as YouTube, Facebook, etc. has emerged. However, the computing power of mobile devices and bandwidth of 2.4 GHz or 5 GHz wireless networks are limited compared to the requirements of high-quality VR. Let us provide some numbers to illustrate this problem. The VR viewport is defined by a device-specific viewing angle (typically 120-degree) that delimits horizontally the scene from head direction center, called the viewport center. To ensure good immersion, the pixel resolution of the displayed viewport is high, typically 4K (3840×2160). Thus, the resolution of the full 360-degreeis at least 12K (11,520 ×6480) [1]. In addition, immersion requires a video frame rate typically around 100 frames per second (fps).Therefore, these specifications are not fully supported in the current market. Existing Head-Mounted Displays (HMDs) on the market are connected via wires to a content server, limiting user action and creating the possibility of entangling. The need to support mobile VR is obvious and has become a challenge to researchers in both academia and industry, prompting great efforts. In [2], the authors proposed a proper down-sampling ratio and quantization parameter method to reduce the bandwidth when streaming and synthesizing the 3DoF+ 360 videos and the authors of [3] investigated tile-based video streaming to reduce bandwidth requirement, while the authors of [4] optimized caching and computation offloading policy to minimize the required average transmission rate under latency and local average energy consumption constraints. In this paper, we propose the concept of 360-degree video streaming architecture for mobile VR. This architecture includes many schemes for reducing bitrate at VR server content, decreasing the computing power as well as optimizing decoding speed at mobile devices to support mobile VR. The proposed architecture was achieved by using the advance of tile-SHVC and mmwave transmission. Tile-SHVC is manually partitioned and extracted for decoding at the mobile device, while PC offloading method is considered to help mobile devices in decoding the VR viewport, and then transmitting the decoded same to the mobile devices through mmWave links.

The rest of this paper is organized as follows: Section 2 gives a brief background of tile-based decoding on mobile device and viewport-based 360-degree video streaming, followed by an overview of mmWave UDP throughput in indoor environments. The novelty of our proposed methods and concept architecture are discussed in Section 3. Section 4 describes the implementation of our method and presents experimental results. Finally, Section 5 draws some conclusions and outlines directions for future works.

## 2. Background

Before explaining the proposed concept architecture, we briefly examine tile-based decoding mechanism and describe the relationship between a VR viewport and tile-SHVC with related technical problems, followed by a discussion of the requirements for implementing mmWave VR with short summary of the solutions available mmWave transmission.

### 2.1. Tiled-HEVC(SHVC) Decoding on Mobile Cores

**Figure 1:**
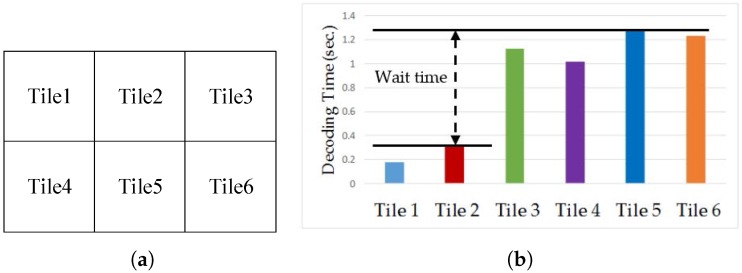
Tile-based HEVC example with (**a**) An example of a frame divided into six tiles, (**b**) Decoding time of each tiles in *PeopleOnStreet* test sequence.

**Figure 2:**
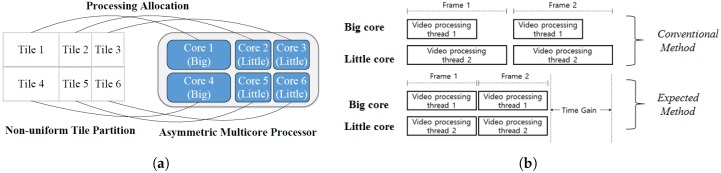
Non-uniform tile partition with (**a**) the concept of tile partition based on Asymmetric Multicore Processor, (**b**) Expected decoding time gain of non-uniform tile partition.

Tile is a new parallel processing tools supported by HEVC as well as SHVC. The frame is partitioned into rectangular regions with flexible horizontal and vertical boundaries, but the boundaries of tiles cannot cross slices. The main purpose of tiles is to enable the use of parallel processing architectures for encoding and decoding. All tiles within a picture are independent from each other except for potential dependencies regarding cross-tile border in-loop filtering. Each tile contains a rectangular arranged group of Coding Tree Units (CTUs) that may have dependencies on the CTUs of other tiles. Figure 1a shows an example of a frame divided into six tiles. Using multiple threads at encoder or decoder to support parallel processing, tile can scale up to asymmetric multicore processors in mobile devices. In which, each core will be assigned to decode specified tiles. Figure 1b shows an example of decoding time for *PeopleOnStreet (3840×2160)* sequence split into six tiles uniformly. In this example, all the tiles have similar decoding complexity because of uniform tile partitioning, but tiles number 1 and number 2 are allocated to big cores that have higher computing power while other tiles are allocated to little cores that have lower computing power. Therefore, Figure 1b shows quite a long gap between the decoding time of tile number 1, tile number 2 and others. The gap of decoding time is the result of not considering the decoding complexity of tiles and computing power of each core. This phenomenon causes a situation in which the thread for a tile with the shortest decoding time waits for the slowest thread even if the fastest thread is completed already causing the decoding efficiency to worsen in the end. Therefore, the decoding time gain can be scaled up by partitioning and allocating non-uniform tiles to suitable cores (e.g., by allocating big tiles to big cores, little tiles to little cores) as shown in Figure 2a to achieve the target decoding time gain as presented in Figure 2b that shows the minimized gap of the decoding time between big and little cores. One of the tile partitioning algorithms is based on the number of bits of CTUs [5]. This algorithm proposes a method that equalizes the total number of bits in each tile to minimize the decoding time between tiles that have many bits or a few bits. However, this research does not consider asymmetric multicore systems.

### 2.2. Viewport-Based 360-Degree Video Streaming

**Figure 3:**
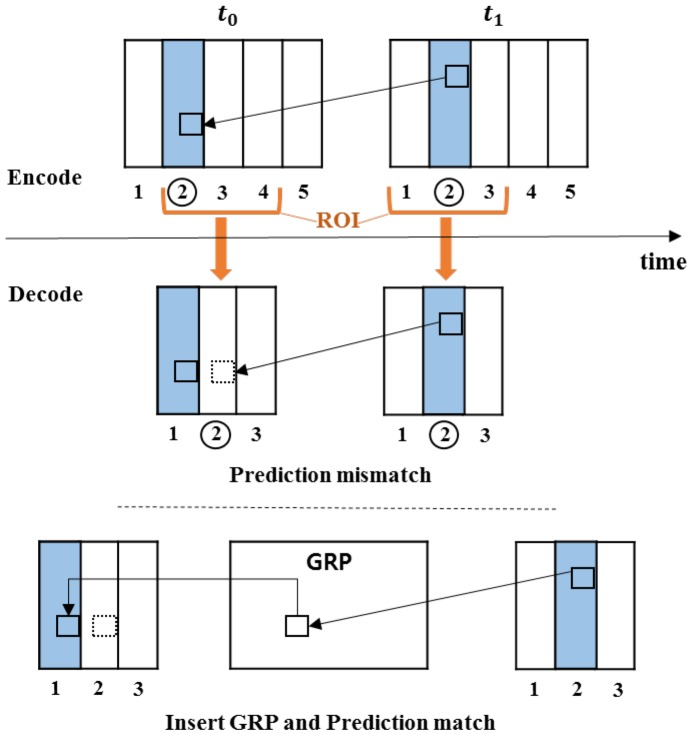
Prediction mismatch and GRP concept for solving a mismatch.

The VR viewport presents a portion of 360-degree video, namely field of view (FOV) [6]. The corresponding FOV is chosen to be transmitted instead of the entire panoramic video, thereby saving bandwidth significantly. This is done based on tiled-SHVC, whose base layer (BL) and enhancement layer (EL) are divided into multiple tiles and only the tiles corresponding to the viewport are streamed. However, when streaming only the corresponding tiles, a prediction mismatch occurs when decoding by referring to the area that is not transmitted. Figure 3 shows the prediction mismatch and its solution. At the encoder, the second tile of the $t_{1}$ picture references the second tile of the $t_{0}$ picture. Considering the viewport, the $t_{0}$ picture transmits the second to fourth tiles, and the $t_{1}$ picture transmits the first to third tiles. The decoder encounters prediction mismatches with reference to the same second tile using the motion vector of the encoder. The authors of [7] proposed a Generated Reference Picture (GRP) to prevent prediction mismatches. All prediction units (PU) of a GRP have an associated motion vector (MV), that compensates for the movement caused by sending only selected tiles. This paper modifies the GRP to fix MV reference errors by using the characteristics of SHVC through the lower layer. However, there is still an overhead in GRP generation.

The moving picture experts group (MPEG) standard leverages motion-constrained tile sets (MCTS) as a classic way of limiting MVs in the current picture [8]. MCTS limits the temporal motion information of the encoder so that the encoding efficiency is slightly reduced. However, a single bitstream using MCTS can decode only the desired tiles of the full picture without additional picture generation. The authors of [9] modified the high-efficiency video coding (HEVC) encoder as a concept of MCTS. Their study was conducted using three tiling methods, resulting in 3% to 6% penalties in compression. However, when only the tile corresponding to the FOV was transmitted, the streaming bitrate saved between 30% and 40%. In contrast, we offer a method for implementing MCTS in SHVC and HEVC. The proposed implementation is adopted using MPEG standard and further presents a method for extracting and decoding selected tiles.

### 2.3. Data Rate Requirement for Mobile VR and mmWave UDP Throughput in Indoor Environments

According to 116th MPEG meeting, the following requirements should be met to support high-quality VR video streaming [1].

- Pixels/degree: 40 (number of pixels per degree).
- Resolution: 11,520 ×6480 (3 times the 4K vertical resolution).
- Frame rate: 90 fps (90 fps can prevent nausea through low delay).
- Environment-based or scene-based audio (360-degree surround sound and object-oriented audio).
- Maximum video and audio delay: 20 ms (time between user interaction and VR image and audio).

**Figure 4:**
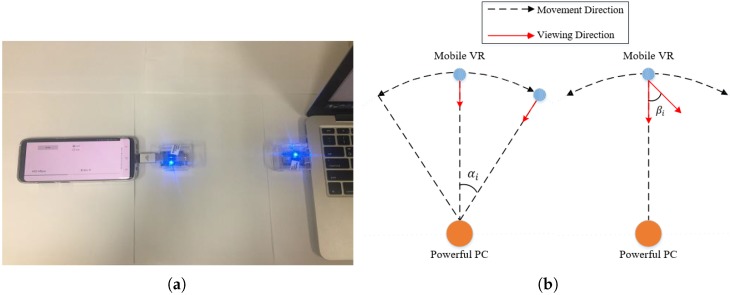
Setting throughput scenario with (**a**) changing the distance, obstacle, (**b**) misalignment.

To satisfy the above requirements, a data rate from0.9546 Gbps to 19.11 Gbps should be supported [10]. Among existing wireless technologies, IEEE 802.11ad which uses the mmWave band (60 GHz) provide above data rate. The major mmWave [11] use cases are intended for indoor video streaming that has used for uncompressed high definition wireless transmission with support for gigabit wireless. The mmWave indoor scenario is characterized by much smaller distances between hosts. In addition, the main factor limiting deployment options is blockages by physical objects such as human bodies. The human body has been shown to cause several signal blockages, reducing the spectral efficiency gains obtained from operations over the wider bandwidths available in mmWave communication, as discussed in [12,13]. Furthermore, the authors of [14] studied peer-to-peer indoor mmWave communications scenarios, under the assumption that random directions of the interferer’s main-lobe. Therefore, directional beams were required to maintain Gb/s links in crowded indoor areas. However, mmWave devices are still under development and there are few that are commercially available. To examine how real-life data transfer rates can be achieved, we used a mmWave VR dongle [15] that acts as a USB 3.0 network adapter with parameters defined in Table 1 to communicate with each other as shown in Figure 4a. In particular, current antennas present 10 dBi of gain, a transmitted equivalent isotropic radiated power (EIRP) of 13 dBm and IEEE 802.11ad single carrier MCS (Modulation and Coding Scheme) of 7. During the test, two mmWave dongles were movable to perform the measurements of parameters when distance, setting an obstacle, and alignment between communicating end points are varied. In these experiments, the receiver continuously receives data from the sender without sending an acknowledgement (ACK) packet.

**Figure 5:**
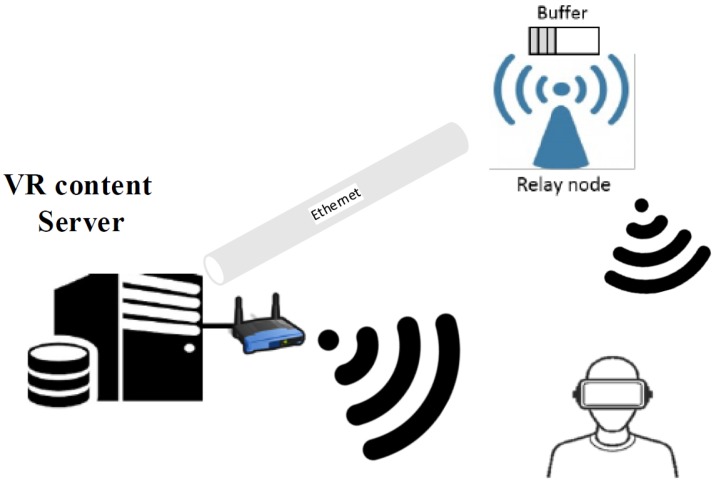
Advances of mmWave VR system using relay node.

As summarized in Table 2, the achieved data transfer rate is approximately 900 Mbps, and not the 4.62 Gbps, as described by IEEE 802.11ad. The fact that the device is connected to a USB port limits the level of power and the kind of antennas this device can receive or mount and the dongle device is still under development and these measurements will have to be revisited in the future when the vendor releases new firmware and device drivers. Better performance could be achieved if the actual issues pertaining to hardware and layer to layer transmission could be overcome. In addition, the data transfer rate decreases as the distance increases, owing to the attenuation of the high frequency signal in the mmWave band. After 3 m, the intensity of the signal suddenly drops and the achievable data rate drops by approximately 38% at around 4 m. During the obstacle test, the achievable data rates fall up to 23% when disturbed by a human head. Also, the achievable data rate reduces according to the misaligned degree $\alpha_{i}$ or $\beta_{i}$ between the transmitter and receiver, as shown in Figure 4b. These experiments indicate that the mmWave channel is quite sensitive to obstacles and misalignments that can suddenly lead to a drop in the bandwidth. They are result of mmWave potential problems including deafness problem, beam misalignment problem, blockage problem [16]. Hence, there is a need to address these problems in the upper layer to improve high-quality VR streaming. In example, using relay node to pre-buffer in case of dropping bandwidth by antenna misalignments like Figure 5.

## 3. Mobile VR: Concept Architecture and Proposed Methods

**Figure 6:**
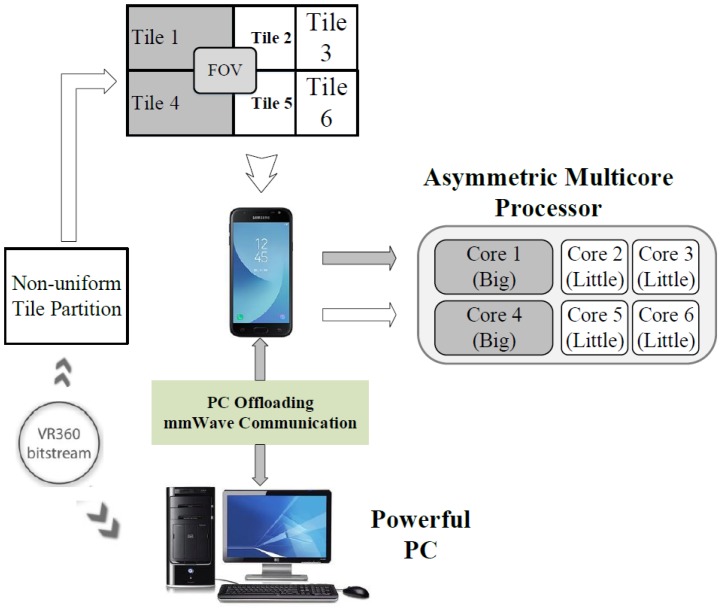
The proposed conceptual architecture.

The goal of proposed architecture is to provide the video of highest quality in limited mobile VR environments, as shown in Figure 6. Through the proposed implementation, the entire BL picture is streamed and rendered in low quality and only the viewport (extracted tiles of the EL picture) is rendered in high quality. The BL picture is received and decoded in parallel at the mobile device, whereas the viewport tiles are decoded on powerful PCs and offloaded to mobile devices via mmWave. Moreover, pictures are tile-based partitioned non-uniformly and allocated to the asymmetric multicore at the mobile device to increase the decoding speed. Overall, concept architecture is based on two main techniques: Tile-based SHVC and PC offloading. This section started by explaining the related tile-SHVC proposes, followed by the PC offloading scheme for delivering the viewport.

### 3.1. Tiled-SHVC for 360-Degree Video Streaming

**Figure 7:**
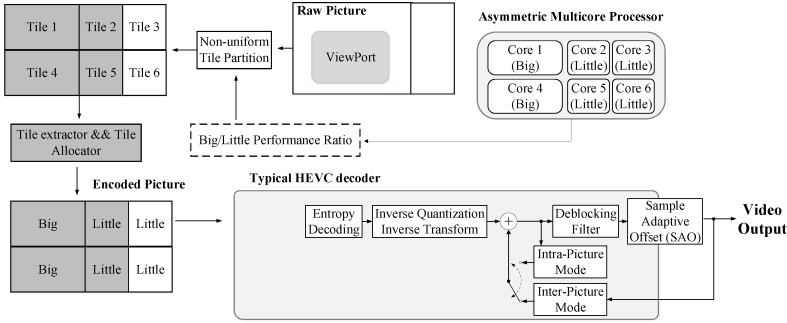
Block diagram of the proposed Tile-SHVC encoding/decoding system.

The functionality of the proposed tiled-based method is described in Figure 7. It includes a tile extractor and tile allocator based on non-uniform tile partition (grey block). As mentioned in Section 2.2, prediction mismatch in the tile extractor occurs when decoding the corresponding tiles because it refers to areas that were not transmitted, the proposed architecture advances the modification of the GRP to solve problems related to undecoded tiles in the two SHVC encoder steps. In addition, we also discuss how to apply MCTS to HEVC encoder.

#### 3.1.1. Challenge: Reference to Undecoded Tiles in the Temporal Inter Prediction (TIP)

**Figure 8:**
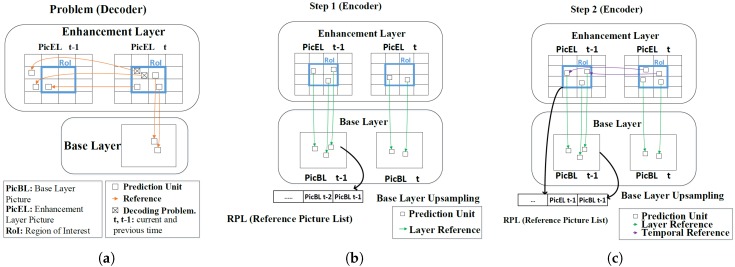
Problem with non-ROI tile references in the SHVC decoder; In Step 1, the EL refers only to the picture upsampled by the BL and in Step 2, the current picture refers to the prediction unit when the TIP points to the tile at the same position in the EL.

The SHVC performs TIP within the same layer and inter layer prediction (ILP) between different layers through an up-sampling filter [17]. This works well when the decoder decodes all layers into a full picture. If only some tiles were decoded, then a problem arises with motion estimation and compensation in TIP. Figure 8a explains an incorrect reference of the problem mentioned above when decoding only viewport tiles in the EL. At that time, the current picture, ($PicEL_{t}$) refers to the previous picture ($PicEL_{t-1}$). If the MV generated by the encoder points to the undecoded tile, the decoding problem occurs. Therefore, the need for correcting the MVs at the encoder in this case is obvious. To handle this problem, we propose a solution includes two processing steps as follows:

- Proposed Step 1: The motion vector of undecoded tile at EL is replaced by the upsampled BL.
  In step 1, we suggest the use of the upsampled PUs at the BL to overcome the problem mentioned in Figure 8a. As shown in Figure 8b, the encoder considers an upsampled PU at the BL as the reference picture, and does not consider the one at the EL. Therefore, the viewport tiles selected at the EL can refer to all areas of the reference picture to eliminate decoding errors at the EL. In addition, the BL covers the entire picture but the EL represents only viewport tiles. However, because the EL does not use TIP, the bitrate increases significantly.
- Proposed Step 2: Available tile encoding in EL using upsampled BL and decoded tile of EL.
  Step 1 solves the problem of referring to the outer region of the viewport. However, as step 1 uses only an upsampled PU at the BL as a reference list, that PUis still available for ILP. Therefore, as shown in Figure 8c, when the MV of the TIP points to a position within the same position tile, the PU of the current picture, ($PicEL_{t}$) refers to the PU of the previous picture, ($PicEL_{t-1}$). When calculating the rate distortion (RD) cost of finding the optimal PU, both the upsampled BL and the previous picture of the EL are included. The encoder chooses the PU with a more efficient RD cost than others from these options. Thus, Step 2 demonstrates an optimized encoding result that is better than Step 1.

#### 3.1.2. Available Tile Encoding for HEVC Decoder Using Intra Prediction

**Figure 9:**
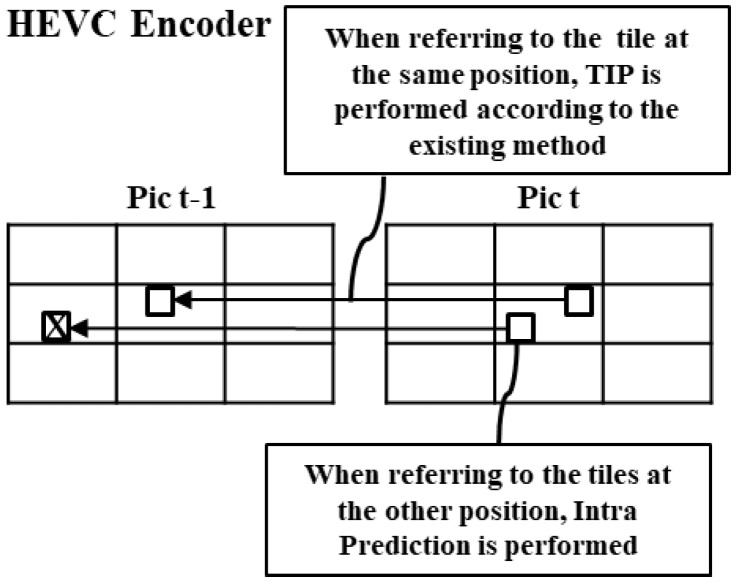
MCTS: TIP perform at the HEVC encoder.

Different from SHVC, the HEVC [18] encoder has a single layer and does not use ILP. Therefore, the HEVC encoder performs intra prediction when the tile temporally references those at the other position as described in Figure 9.

#### 3.1.3. Implementation of Tiled Extractor by Modifying the TIP Information

**Figure 10:**
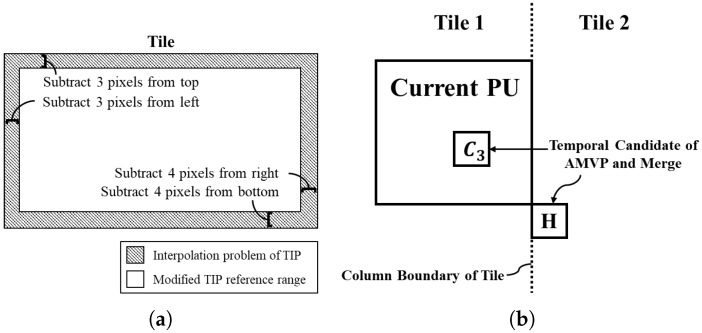
Tile Extractor Implementation Problems with (**a**) Interpolation problem of referring to a tile at the same position in the TIP, (**b**) Temporal candidate problem at column boundary between tiles.

When the reference pictures have already decoded, the tiles are not temporally independent even the tiles are spatially independent to support parallel processing [19]. Therefore, interpolation should be considered when using MVs to determine if the referenced PU is within the tile at the same position in the TIP. Both HEVC and SHVC use an *eight-tap* filter to interpolate luma prediction. When the *eight-tap* filter is applied horizontally, three pixels to the left and four pixels to the right of the current pixel are used. When applied vertically, the top three pixels and the bottom four pixels from the current pixel are used. Figure 10a describes the interpolation problem of referring to the tile at the same TIP position. If the PU temporally references the area with the interpolation problem, the tile cannot be transmitted independently because the PU interpolates pixels from other tiles. Therefore, the oblique area should be excluded from the TIP reference range. When implementing MCTS in SHM [20] and HM [21], the position of the current PU should be considered. The *x* and *y* pixel values at the top and left of the current PU can be obtained using the *getCUPelX()* and *getCUPelY()* functions, while the *x* and *y* pixel values at the bottom and right can be obtained by adding the values obtained from the *getWidth()* and *getHeight()* functions in the HM and SHM software. However, if the current PU is not in the *2N × 2N* mode, its position should be changed. Because the four functions discussed above return a value based on the *2N × 2N* mode, the position and size of the PU can be obtained by considering the position of the PU in eight partition modes.

The HEVC and SHVC encoders use advanced MV prediction (AMVP) and merge to reduce the amount of motion information in the inter prediction. Both modes use spatial and temporal candidate blocks. As mentioned in Section 2.2, temporal candidates should be considered for MCTS implementation. The block at the bottom right and at the center of the current PU are used as temporal candidates [22]. Thus, when the block to the bottom right of the current PU belongs to a CTU beyond the current CTU row, the block is not considered as a temporal candidate. However, there is a problem when the candidate block goes out of the column boundary, and not the CTU row. Figure 10b describes the temporal candidate problem at the column boundary between tiles. When the *H* candidate block is selected, independent tile transmission is not guaranteed because it uses motion information from another tile. The modified HM and SHM first determines whether the current CTU is located on the right side of the tile, that is obtained using the *getRightEdgePosInCtus()* function. The current CTU position is obtained using the *getFrameWidthInCtus()* and the *getCtuRsAddr()* functions. Based on whether the current PU in the CTU is on the right side of the CTU, the position of the current PU is obtained using *deriveRightBottomIdx()*. The *getNumPartInCtuWidth()* and the *getNumPartInCtuHeight()* functions are used to determine if the current PU position is on the right side of the CTU. If both conditions are met, the *H* block is excluded from the candidate.

### 3.2. Proposed Tiled-HEVC Partitioning for Mobile Devices

**Figure 11:**
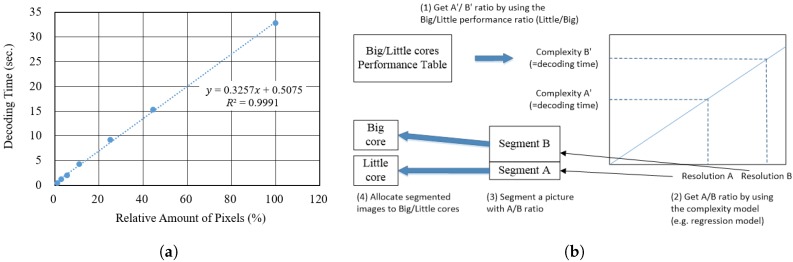
Tile partitioning–based HEVC parallel decoding optimization for asymmetric multicore processors with (**a**) Regression model indicating a correlation between resolutions and decoding complexity, (**b**) The procedure of the proposed non-uniform tile partitioning method.

The decoding complexity of video pictures is affected by many explicit factors (e.g., resolution and quantization parameter (QP)) and implicit others [23]. As discussed in Section 2.1, we proposed a new tile partitioning method based on the decoding complexity predicted from the resolution of tiles and the performance ratio of the big and little cores. The method optimizes the decoding time of a video sequence by partitioning and allocating non-uniform tiles to suitable cores (e.g., by allocating big tiles to big cores, little tiles to little cores). The proposed method is based on a regression model that indicates a correlation between the resolutions and decoding complexity (decoding time) [24,25,26]. To build this model, we used *PeopleOnStreet* that have a resolution of 3840×2160, QP 22, and random access (RA) coding structure as a test sequence. The regression model is shown in Figure 11a. The X-axis indicates the relative number of pixels. For example, 100% in X-axis means 8,294,400 pixels of 3840×2160. Similarly, the 80% and 60% mean 6,635,520 and 4,976,640 down-sampled pixels, respectively. The Y-axis indicates the decoding time of the test sequences with multiple resolutions. Based on the regression model, we can obtain the decoding complexity from a tile with a given resolution as follows:

(1) $$\mathrm{Comp}=C_{1}\mathrm{Res}+C_{2}$$

where *Res* is the resolution of the tile, $C_{1}$ and $C_{2}$ are the coefficients and *Comp* is the decoding complexity of the tile. The computed complexity *Comp* is given by (1). In addition, we can calculate the decoding complexity ratio between the big and little tiles for the big and little cores as:

(2) $${\mathrm{RatioComp}}_{LB}=\frac{{\mathrm{Comp}}_{L}}{{\mathrm{Comp}}_{B}}$$

where ${\mathrm{Comp}}_{B}$ and ${\mathrm{Comp}}_{L}$ are the complexities of the big and little tiles, respectively. The ${\mathrm{RatioComp}}_{L}B$ is given from performance ratio between the big and little cores, and it depends on decoding systems with asymmetric multicores. Thus, we obtain:

(3) $${\mathrm{RatioComp}}_{LB}=\frac{C_{1}{\mathrm{Res}}_{L}+C_{2}}{C_{1}{\mathrm{Res}}_{B}+C_{2}}$$

where ${\mathrm{Res}}_{B}$ and ${\mathrm{Res}}_{L}$ are the resolutions of the big and little tiles, respectively. If the total resolution of a frame is 100, we obtain:

(4) $$100=N_{B}{\mathrm{Res}}_{B}+N_{L}{\mathrm{Res}}_{L}$$

where $N_{B}$ and $N_{L}$ are the number of the big and little cores, respectively. When we substitute (5) and (6) into (3), we get the following equations:

(5) $${\mathrm{Res}}_{L}=\frac{\left(100-N_{B}{\mathrm{Res}}_{B}\right)}{N_{L}}$$

(6) $${\mathrm{Res}}_{B}=\frac{\left(100-N_{L}{\mathrm{Res}}_{L}\right)}{N_{B}}$$

(7) $${\mathrm{Res}}_{B}=\frac{100C_{1}+N_{L}C_{2}\left(1-{\mathrm{RatioComp}}_{LB}\right)}{C_{1}\left(N_{L}{\mathrm{RatioComp}}_{LB}+N_{B}\right)}$$

(8) $${\mathrm{Res}}_{L}=\frac{{\mathrm{RatioComp}}_{LB}\left(N_{B}C_{2}+100C_{1}\right)-N_{B}C_{2}}{C_{1}\left(N_{L}{\mathrm{RatioComp}}_{LB}+N_{B}\right)}$$

Finally, if we obtain the regression model, the performance ratio between big and little cores, and the number of big and little cores, we can calculate ${\mathrm{Res}}_{B}$ and ${\mathrm{Res}}_{L}$ as in (7) and (8). Figure 11b shows the procedure for the proposed method, that (i) calculates the ratio between the decoding complexities of A′ and B′ based on the performance ratio of big and little cores, (ii) obtains resolutions of tiles using the complexity–resolution regression model, (7) and (8), (iii) segments a picture into non-uniform tiles, and (iv) allocates segmented tiles to the big and little cores.

### 3.3. PC Offloading over an mmWave Connection

**Figure 12:**
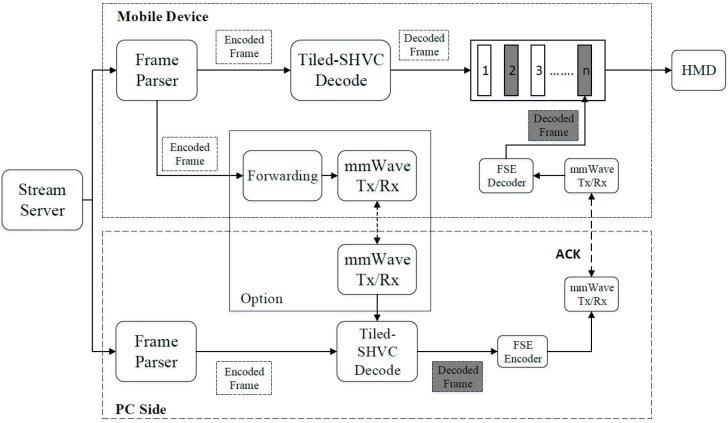
The PC offloading system and the first synchronization method.

In MVP, the viewport is extracted and encoded at the EL. To overcome the limitations in power and computation for handling the viewport at high resolution, MVP uses PC offloading to decode the viewport and transmit the decoded viewport to mobile devices using mmWave connections. The main idea behind the proposed scheme is as follows:

- To solve the high resolution of 360-degree viewport streaming. Instead of other wireless 802.11 technologies, the mmWave link is applied to support high speed.
- To avoid the overflow issue or quite low performance issue of high-resolution video processing in mobile devices in terms of decoding rate [27], the decoding task is offloaded to a powerful PC. Figure 12 shows the proposed system with an offloading mechanism. This mechanism helps mobile devices reducing the computation and power required for a decoding process. The PC could receive encoded bitstream from a server or directly from the mobile devices to decode and transmit the decoded viewport to mobile device over mmWave links.
- As mentioned in Section 2.3, to reduce the effect of deafness, the beam misalignment and blockage problems, this study implements synchronization mechanisms to ensure the performance of the connected link. When data packets drop, the ACK packets are fed back from the mobile device to the PC, confirming successfully received packets. Next, ACK is completed on the PC that then sends the next packet to the mobile device. If the confirmation fails, the PC will re-send the current packet until it succeeds, or timeout is reached.

**Figure 13:**
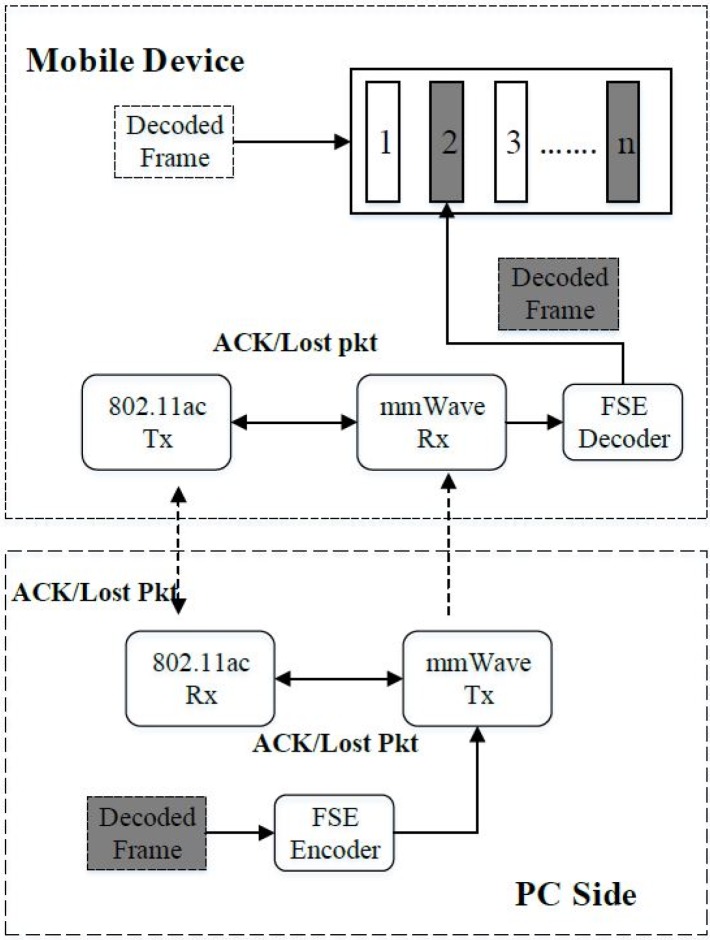
Second proposed for synchronization method.

The first synchronization mechanism is implemented as a classic method of creating a reliable session by sending ACK messages and lost packets on mmWave channels as shown in Figure 12. However, feedback packets reduce the data rate by waiting for ACK packets and re-sending lost packets. Therefore, this paper further introduces a second synchronization mechanism that uses TCP 802.11ac channel for sending ACK packets from the mobile device to the PC and lost packets from the PC to the mobile devices as shown in Figure 13.

## 4. Experimental Results and Discussion

In this section, the proposed method is implemented and evaluated through several unit tests.

### 4.1. Performance Evaluation of Tiled-SHVC Extractor for VR Streaming

This experiment uses test sequences selected by the Joint Video Expert Team (JVET), as shown in Table 3. Test sequences are encoded with general coding options for random access (RA) coding structure as shown in Table 4.

Table 5 and Table 6 show the increased bitrate and decreased peak signal-to-noise ratio (PSNR), respectively, compared to the original encoding. The bitrate of the modified SHM and HM increases by 8% and 11% on average. The PSNR of the modified SHM and HM decreases between 0.04 and 0.05 dB on average. The proposed method increases the bitrate and decreases the PSNR because the motion vector, temporal candidates of AMVP and merge are limited to allowing tiles to be transmitted independently. Table 7 shows the approximate ratios of the bitrate when a selected tile is transmitted using the proposed SHM and HM encoding compared to the existing encoding technique. When the encoder independently transmits tiles corresponding to the viewport through the proposed SHM encoder, average bitrate savings of 51% and 87% are achieved for four tiles and one tile, respectively. For the proposed HM encoder, average bitrate savings of 49% and 86% are achieved. Using the proposed encoding, the bitrate is significantly reduced when the server transmits only some tiles of the entire picture. The demonstration of this method is shown in section of PC offloading performance evaluation.

### 4.2. Performance Evaluation of Non-Uniform Tile Partitioning on Mobile Core

**Figure 14:**
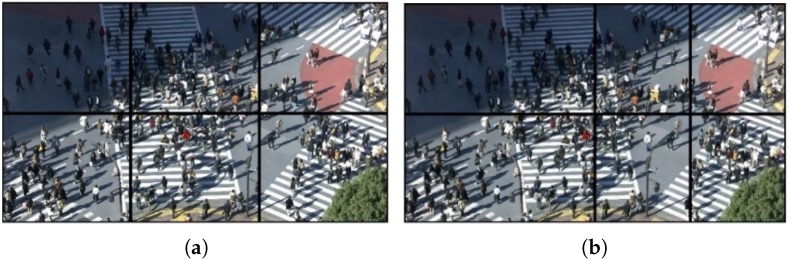
Tile partition of *PeopleOnStreet* test sequence with (**a**) conventional uniform tile partitioning, (**b**) Proposed non-uniform tile partitioning method considering Samsung Galaxy S7 Edge environments.

**Figure 15:**
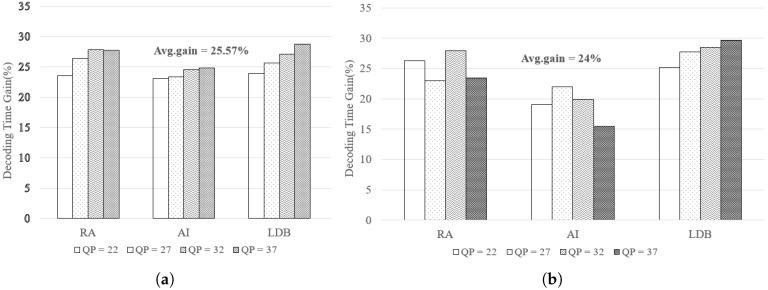
Decoding Time Gain (%) following test sequences (**a**) *PeopleOnStreet*, (**b**) *Traffic*.

**Figure 16:**
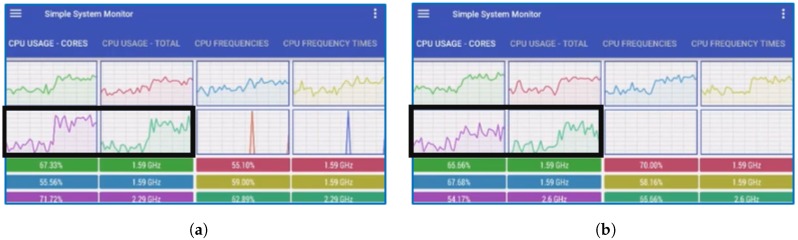
Comparison of the big cores use rates between the conventional and proposed tile partitioning methods.

In this work, we used the HM15.0 encoder and two 4K test sequences (*PeopleOnStreet* (3840×2160) and *Traffic* (3840×2048)) for encoding. Table 8 presents the encoding options. During the encoding step, the internal option *TileUniformSpacing* is set to value the ‘0’ for non-uniform tile partitioning. The *TileColumnWidthArray* and *TileRowHeightArray* options are used to adjust the resolutions of each tile. We executed this experiment using the conventional and proposed methods, as shown in Figure 14. For real-time decoding, open source OpenHEVC decoder was used that provides an Android development branch [28]. We modified a function in the OpenHEVC decoder named *hls_decode_entry_tiles* to implement the proposed method. In addition, a function named *sched_setaffinity* was used to allocate video decoding threads to the big and little cores. The Samsung Galaxy S7 Edge (Samsung Electronics, made in Vietnam) has four big and four little cores, but this experiment considers only two big and four little cores because two big cores are always on online state while other two big cores are on offline state usually for power saving. Figure 15 shows the experimental results. The test results show that the proposed tile partitioning method achieves a decoding time gain of up to 25% compared to the uniform tie partitioning method. In addition, Figure 16 shows the increased use of big cores when the proposed optimal tile partitioning is applied. In Figure 16a, the larger fluctuations show that the big cores wait for little cores to complete decoding a picture, although the big cores had already completed decoding its assigned tile. Conversely, Figure 16b shows relatively stable use rates of big cores compared to the left figure. This is because the proposed tile partition method minimizes the wait time of the big cores. The minimized wait time enhances the overall decoding performance.

### 4.3. Performance Evaluation of PC Offloading for VR Streaming

This subsection explains how the decoded frame and viewport is transmitted to mobile devices over mmWave.

#### 4.3.1. PC Offloading Scenario

To verify the proposed approach, we implemented a practical testbed as shown in Figure 20. The set-up information is listed in Table 9.

**Figure 17:**
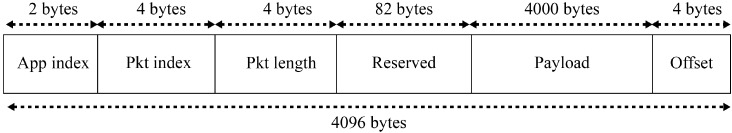
Packet structure of the mmWave connection.

During the transaction, the decoded pictures are divided into small segments of 4000 bytes and assigned to a mmWave packet stream to a mobile device. The packet structure of the application layer is illustrated in Figure 17. A packet intended for video streaming is designed with an “app index” and a “pkt index” to number packets and cover the synchronization issue using a synchronization mechanism. Each data packet contains one identification number for indexing. The PC sequentially sends packets to the mobile VR device via mmWave links. With the first synchronization method, the PC waits for an ACK message before sending the next packet or sending lost packets. However, with the second synchronization sends packets to the mobile VR device via mmWave links without waiting for an ACK packet. The received index is used to build an ACK packet on the mobile VR device when it detects packet loss and the mobile VR sends an ACK packet to the PC via the TCP 802.11ac interface. The mobile VR receives packets from a TCP socket and inserts them correctly into the buffer. The “pkt length” is the length of the payload. It must be a multiple of 4096 bytes because the unit in RF transactions is 4096 bytes. The payload will be divided into 4k packets per transaction. Each packet may be dropped or repeated during the RF transaction. The payload contains video data, either encoded or decoded video data. The final four bytes are “offset” bytes used for synchronization.

#### 4.3.2. Proposed Synchronization Method Performance

**Figure 18:**
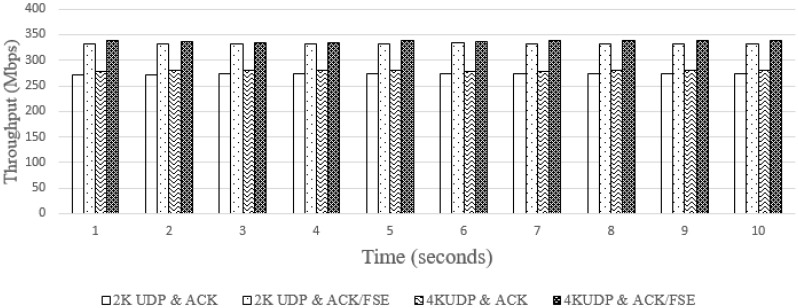
End-to-end throughput with mmWave ACK/FSE.

According to the proposed methods in Figure 12 and Figure 13, we implemented both proposed synchronization methods for offloading video. To downsize raw data, we implemented a lossless entropy coding named Finite State Entropy (FSE) [29] and that yielded an impressive maximum result of 40% reduction in data size. Figure 18 shows the results of PC offloading with using the first synchronization method. The end-to-end throughput was approximately 300 Mbps, for both the 2K and 4K resolutions video. We apply the FSE coder to gain a higher throughput of approximately 350 Mbps. The number of retransmitted packets for the 2K and 4K video in this case was 4.76% and 9.84%, respectively. Hence, the second synchronization method was designed to enhance the performance of the first method by using a TCP 802.11ac wireless to send the transmitted packets.

**Figure 19:**
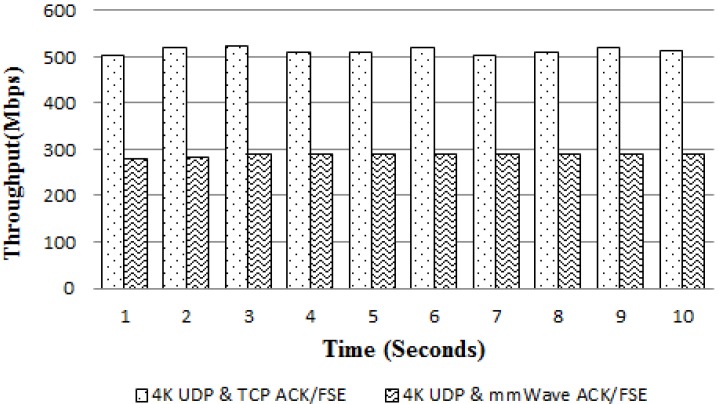
End-to-end throughput comparison between mmWave ACK and TCP ACK.

**Figure 20:**
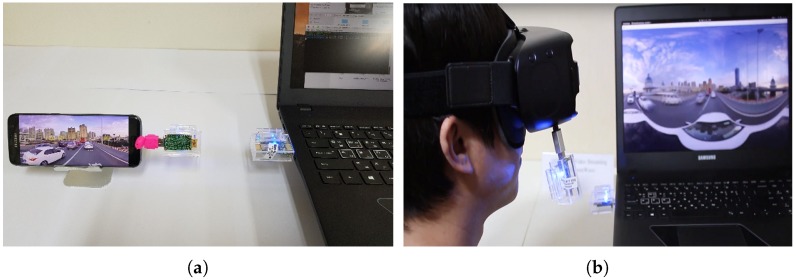
Implemented mobile VR 360-degree video player using mmWave communication; (**a**) 3840×1920 full picture consisting of 9 tiles; (**b**) one extracted tile of 9 tiles.

As proof of our assumption, Figure 19 shows the performance of the PC offloading via the second synchronization method. By reducing the waiting time for the ACK messages and re-sending lost packets, the second method offers a significant improvement in throughput by 180%, leading to the achievement of a throughput of 500 Mbps. The quality of the 4K resolution video on the server side (i.e., powerful PC) and client (i.e., mobile device) was determined as shown in Figure 20. In this demonstration, the raw video (or viewport only) after decoding is transmitted over the mmWave session. The 4K video results shown in Figure 20b confirm that the display quality is quite good for 4K resolution videos on mobile VRs.

## 5. Conclusions

This paper proposed a conceptual architecture for mobile VR streaming, applying advanced tiled-SHVC and mmWave communication. This architecture uses a tile extractor to independently transmit tiles. The drawback of the EL tile extraction was solved by adopting upsampled BLs. This mechanism had a slightly lower HM encoding efficiency average bitrate (i.e., 11%) and a 0.05 dB PSNR over the original encoding because the MV and temporal candidates of the AMVP and merge were limited. However, SHM and HM saved an average of 48% and 47% bitrates when transmitting four ROI tiles among nine other tiles. In addition, SHM and HM saved at least 49% up to 87% of the bitrate with four sequence averages when only one tile was sent. Moreover, we presented a novel tile partitioning method for parallel decoding on asymmetric multicores. The size of the non-uniform tiles is based on the regression model of the computational complexity per video resolution. This contributed to a 25% speeding up of the decoding process. This paper also proposed PC offloading mechanisms using mmWave communication. allowing PC decoding full video (or viewport only) and raw data forwarding to mobile devices over the mmWave connections. Two synchronization schemes were applied to reduce the effect of packet loss and fluctuating mmWave channels. In the future, we will continue to develop the concept architecture by merging the currently achievable results and considering relay prototypes to support blockage cases for mmWave communications.

References yes

## Figures and Tables

**Table 1:** mmWave antenna specifications.

Parameters	Type	Unit
*Radiation type*	Enfire/Broadside	
*Polarization*	Linear	
*2Tx Array Peak Gain*	10	dBi
*2Rx Array Peak Gain*	10	degree
*3 dB Beamwidth @co-plane(include BF)*	110	degree

**Table 2:** UDP throughput vs. distance, obstacle, and misalignment of dongle USB 3.0 mmWave link.

**Distance**	0.5 m	1 m	1.5 m	2 m	2.5 m	3 m	3.5 m	4 m
	928.5 Mbps	922.6 Mbps	893.7 Mbps	889.6 Mbps	883.4 Mbps	748.4 Mbps	670.5 Mbps	577.7 Mbps
**Obstacle**	Obstacle: Book		Obstacle: Hand			Obstacle: Head		
	828.7 Mbps		875.6 Mbps			685.6 Mbps		
**Beam Align**	0°		15°		25°		35°	
	693.3 Mbps		706.7 Mbps		657.4 Mbps		275.1 Mbps	

**Table 3:** Information of test sequences.

Name	Resolution	Frame Length	Frame Rate
KiteFlite	8192 × 4096	300	30 fps
Harbor	8192 ×4096	300	30 fps
Trolley	8192 ×4096	300	30 fps
GasLamp	8192 ×4096	300	30 fps

**Table 4:** Coding options.

Coding Option	SHM Parameter	HM Parameter
Version	12.3	16.16
CTU size	64 × 64	
Coding structure	RA	
QP	**-**	22
BL QP	22	**-**
EL QP	22	**-**
Tile	Uniformly 3 × 3 = 9 tiles	
Slice Mode	Disable all slice options	
WPP mode	Disable all wpp options	

**Table 5:** Bitrate increase ratio compared to original encoding.

Name	Modified SHM	Modified HM
KiteFlite	6%	8%
Harbor	5%	8%
Trolley	10%	13%
GasLamp	13%	17%
Average bitrate increase	8%	11%

**Table 6:** PSNR decrease compared to the original encoding.

Name	Modified SHM	Modified HM
KiteFlite	−0.04 dB	−0.05 dB
Harbor	−0.03 dB	−0.02 dB
Trolley	−0.05 dB	−0.07 dB
GasLamp	−0.05 dB	−0.06 dB
Average PSNR increase	−0.04 dB	−0.05 dB

**Table 7:** Comparison ratio of bitrate to select and transmit tiles using modified SHM and HM encoding.

Name	HM		SHM	
	4 Tiles Bitrate Saving (apx)	1 Tile Bitrate Saving (apx)	4 Tiles Bitrate Saving (apx)	1 Tile Bitrate Saving (apx)
KiteFlite	51%	87%	52%	88%
Harbor	51%	87%	53%	88%
Trolley	49%	87%	50%	87%
GasLamp	47%	86%	49%	87%
Average bitrate saving	*49%*	*86%*	*51%*	*87%*

**Table 8:** General coding options.

Coding Options	Parameters
Coding Structure	RA (Random Access) AI (All Intra) LDB (Low-Delay B)
QP	22,27,32,37
Number of Tiles	6 (3 × 2)

**Table 9:** Setting parameters for PC offloading scenario.

**Mobile device**	Samsung GalaxyS7
**PC**	Core-i7/4 cores
**mmWave device**	Wigig USB3.0 Dongle
**Distance**	1 m
**SHVC bitstream**	DrivingInCity (3840 × 1920_1920 × 1080)

## References

[1] Champel M., Stockhammer T., Fautier T., Thomas E., Koenen R. Quality Requirements for VR. Proceedings of the 116th MPEG Meeting of ISO/IEC JTC1/SC29/WG11, MPEG 116/m39532.

[2] Jeong J., Jang D., Son J., Ryu E.S. (2018). 3DoF+ 360 Video Location-Based Asymmetric Down-Sampling for View Synthesis to Immersive VR Video Streaming. Sensors.

[3] Gaddam V.R., Riegler M., Eg R., Griwodz C., Halvorsen P. (2016). Tiling in interactive panoramic video: Approaches and evaluation. IEEE Trans. Multimedia.

[4] Sun Y., Chen Z., Tao M., Liu H. (2018). Communication, Computing and Caching for Mobile VR Delivery: Modeling and Trade-off. arXiv.

[5] Baik H., Song H. A complexity-based adaptive tile partitioning algorithm for HEVC decoder parallelization. Proceedings of the 2015 IEEE International Conference on Image Processing (ICIP).

[6] Reichelt S., Häussler R., Fütterer G., Leister N. (2010). Depth cues in human visual perception and their realization in 3D displays. Proceedings of the Three-Dimensional Imaging, Visualization, and Display 2010 and Display Technologies and Applications for Defense, Security, and Avionics IV.

[7] Sánchez Y., Skupin R., Schierl T. Compressed domain video processing for tile based panoramic streaming using HEVC. Proceedings of the 2015 IEEE International Conference on Image Processing (ICIP).

[8] Wang Y.K., Hendry M.K. Viewport dependent processing in VR: Partial video decoding. Proceedings of the 116th MPEG Meeting of ISO/IEC JTC1/SC29/ WG11, MPEG 116/ m38559.

[9] Zare A., Aminlou A., Hannuksela M.M., Gabbouj M. (2016). HEVC-compliant tile-based streaming of panoramic video for virtual reality applications. Proceedings of the 2016 ACM on Multimedia Conference.

[10] Qi Y., Hunukumbure M., Nekovee M., Lorca J., Sgardoni V. Quantifying data rate and bandwidth requirements for immersive 5G experience. Proceedings of the 2016 IEEE International Conference on Communications Workshops (ICC).

[11] Nitsche T., Cordeiro C., Flores A.B., Knightly E.W., Perahia E., Widmer J.C. (2014). IEEE 802.11 ad: Directional 60 GHz communication for multi-Gigabit-per-second Wi-Fi. IEEE Commun. Mag..

[12] Lu J.S., Steinbach D., Cabrol P., Pietraski P. (2012). Modeling human blockers in millimeter wave radio links. ZTE Commun..

[13] Ramirez D., Huang L., Wang Y., Aazhang B. (2017). On opportunistic mmWave networks with blockage. IEEE J. Sel. Areas Commun..

[14] Gapeyenko M., Samuylov A., Gerasimenko M., Moltchanov D., Singh S., Aryafar E., Yeh S.P., Himayat N., Andreev S., Koucheryavy Y. Analysis of human-body blockage in urban millimeter-wave cellular communications. Proceedings of the 2016 IEEE International Conference on Communications (ICC).

[15] Seed Studio. https://www.seeedstudio.com/WiGig-USB3.0-Dongle-p-2827.html.

[16] Shokri-Ghadikolaei H., Fischione C., Popovski P., Zorzi M. (2016). Design aspects of short-range millimeter-wave networks: A MAC layer perspective. IEEE Netw..

[17] Boyce J.M., Ye Y., Chen J., Ramasubramonian A.K. (2016). Overview of SHVC: Scalable extensions of the high efficiency video coding standard. IEEE Trans. Circuits Syst. Video Technol..

[18] Sullivan G.J., Ohm J.R., Han W.J., Wiegand T. (2012). Overview of the high efficiency video coding (HEVC) standard. IEEE Trans. Circuits Syst. Video Technol..

[19] Misra K., Segall A., Horowitz M., Xu S., Fuldseth A., Zhou M. (2013). An overview of tiles in HEVC. IEEE J. Sel. Top. Signal Process..

[20] JCT-VC. https://hevc.hhi.fraunhofer.de/shvc.

[21] JCT-VC. https://hevc.hhi.fraunhofer.de/.

[22] Feldmann C., Bulla C., Cellarius B. Efficient stream-reassembling for video conferencing applications using tiles in HEVC. Proceedings of the International Conferences on Advances in Multimedia (MMEDIA).

[23] Bossen F., Bross B., Suhring K., Flynn D. (2012). HEVC complexity and implementation analysis. IEEE Trans. Circuits Syst. Video Technol..

[24] Roh H.J., Ryu Y., Ryu E.S. Video Decoding Complexity Analysis Based on HEVC Resolution. Proceedings of the Fall Conference of Korea Information Processing Society (KIPS).

[25] Ryu Y., Roh H.J., Kang S.J., Kim S.K., Ryu E.S. Non-Uniform HEVC Tile Partitioning Method for Asymmetric Multicores. Proceedings of the 11th Asia Pacific International Conference on Information Science and Technology (APIC-IST 2016).

[26] Ryu Y., Roh H.J., Ryu E.S. (2016). Tile Partitioning-based HEVC Parallel Decoding Optimization for Asymmetric Multicore Processor. J. Korean Inst. Inf. Sci. Eng..

[27] Van Dien N., Ryu E.S. Performance comparison of SIMD-based HEVC decoders on mobile processor. Proceedings of the 2017 International Conference on Information and Communications (ICIC).

[28] OpenHEVC. https://github.com/OpenHEVC/openHEVC.

[29] Zstandard F. Zstandard. http://facebook.github.io/zstd/.

